# Determining Vertical Dimension in Edentulous Patients Through Cephalometric Evaluation: A Cross-Sectional Study

**DOI:** 10.7759/cureus.88959

**Published:** 2025-07-29

**Authors:** Chamanthi P, HimaBindu G, Raja Reddy N, Venkata Saiteja Mungara, Hemanth Kumar G, Keerthi Priya Sanam

**Affiliations:** 1 Department of Prosthodontics, Chadalawada Krishna Srinivasa Teja Institute of Dental Sciences and Research, Tirupati, IND; 2 Department of Prosthodontics, Narayana Dental College and Hospital, Nellore, IND

**Keywords:** cephalometric analysis, complete denture, edentulous patients, facial proportions, vertical dimension

## Abstract

Introduction

Accurate determination of occlusal vertical dimension (OVD) is essential for the success of complete denture prostheses, as it influences esthetics, phonetics, comfort, and function. Traditional methods for assessing OVD often lack standardization and can be inconsistent, particularly in completely edentulous patients. Cephalometric analysis offers a radiographic alternative based on stable craniofacial landmarks. This study aimed to evaluate the reliability of cephalometric landmarks, both hard and soft tissue, angular parameters, and mandibular length, in determining vertical dimension in edentulous patients with and without dentures.

Methods

A cross-sectional in vivo study was conducted on 40 completely edentulous individuals. Standardized digital lateral cephalograms were recorded in two stages: without dentures (WOD) at physiologic rest and with dentures (WD) in centric occlusion. Five cephalometric parameters were assessed using digital cephalometric analysis software: Anterior Nasal Spine-Menton (ANS-Me) representing hard tissue, Subnasale-Menton (Sn-Me) representing soft tissue, Nasion-Facial Centre-A-point (N-Fc-A) representing the middle third facial angle, Anterior Nasal Spine-Xi-Suprapogonion (ANS-Xi-Pm) representing the lower third facial angle, and Condylion-Gnathion (Co-Gn) representing mandibular length. Conventional vertical dimension was recorded using Niswonger’s method and Silverman’s closest speaking space technique. Statistical analysis was performed using paired t-tests and Pearson’s correlation coefficient with SPSS software, version 25.0 (IBM Corp., Armonk, NY, USA).

Results

Cephalometric values demonstrated strong positive correlations between without dentures (WOD) and with dentures (WD) conditions, particularly for soft tissue proportion (r = 0.928), middle third facial angle (r = 0.923), and hard tissue proportion (r = 0.870), with no statistically significant differences (p > 0.05). Interocclusal clearance remained consistent across all methods.

Conclusion

Cephalometric analysis provides a reliable, objective, and reproducible adjunct for occlusal vertical dimension (OVD) determination in edentulous patients. Its integration into digital prosthodontics may enhance accuracy and streamline denture fabrication, especially in complex or compromised clinical cases.

## Introduction

The stomatognathic system is a complex and adaptable structure that requires harmony among muscles, bones, joints, and teeth [1]. Complete denture prosthodontics aims to restore lost dentition and associated structures, emphasizing phonetics, esthetics, function, and health [2]. A critical aspect of denture fabrication is establishing the correct vertical dimension (VD), defined as the facial height maintained either by tooth contact or muscular tone [3]. Vertical dimension at occlusion (VDO) refers to the distance between two reference points when teeth are in maximal inter-cuspal position, while vertical dimension at rest (VDR) is recorded when the mandible is in a physiologic rest position [4].

Maintaining optimal VD ensures appropriate freeway space, aesthetic balance, and muscular efficiency [5]. Deviations from the ideal VD may result in aesthetic concerns, discomfort, and functional impairments . Proper VD is essential for mastication, phonation, esthetics, and temporomandibular joint function [6]. Despite the availability of various mechanical and physiological methods, no universal standard for VD determination exists due to individual variation [4].

Cephalometric analysis offers a radiographic approach for evaluating craniofacial proportions using stable anatomical landmarks [7,8]. The advent of digital software has enhanced the accuracy and efficiency of cephalometric measurements [1]. This study aims to evaluate whether cephalometric analysis of hard and soft tissue landmarks correlates reliably with conventional clinical methods for determining VDO in edentulous patients.

## Materials and methods

Study design

With prior clearance from the Institutional Ethics Committee (approval no: CKS/Prostho/17-18/004), this in vivo observational study was conducted in the Department of Prosthodontics at Chadalawada Krishna Srinivasa Teja Institute of Dental Sciences and Research, Tirupati, Andhra Pradesh, India. The study adhered to the principles outlined in the Declaration of Helsinki. After receiving a detailed description of the study’s purpose and procedures, all participants voluntarily provided written informed consent.

Sample size determination

The sample size calculation was based on pilot data assessing vertical dimension differences with and without dentures using cephalometric analysis. Assuming a mean difference of 0.9 mm and a standard deviation of 1.5 mm, a minimum of 38 subjects was required to achieve a power of 80% at a 95% confidence interval. To account for potential attrition or data inconsistencies, the sample size was increased to 40 participants. The sample size was calculated using the formula:

n= Z^2^_α/2_ . σ2/ d2

where: Zα/2​ = 1.96 for a 95% confidence level, σ = standard deviation from a previous pilot study/related literature, and d = acceptable margin of error.

Participant selection

A total of 40 completely edentulous patients, including both males and females, were recruited irrespective of age. Inclusion criteria comprised patients with a skeletal Class I relationship and without systemic conditions that could affect bone metabolism, such as diabetes mellitus, thyroid disorders, osteoporosis, or chronic kidney disease. Patients with facial asymmetry, maxillofacial trauma, congenital or acquired craniofacial anomalies, prior orthognathic or reconstructive surgery, or symptoms of temporomandibular joint dysfunction were excluded. Individuals unable or unwilling to undergo radiographic evaluation were also excluded.

Establishment of the vertical dimension

**Figure 1:**
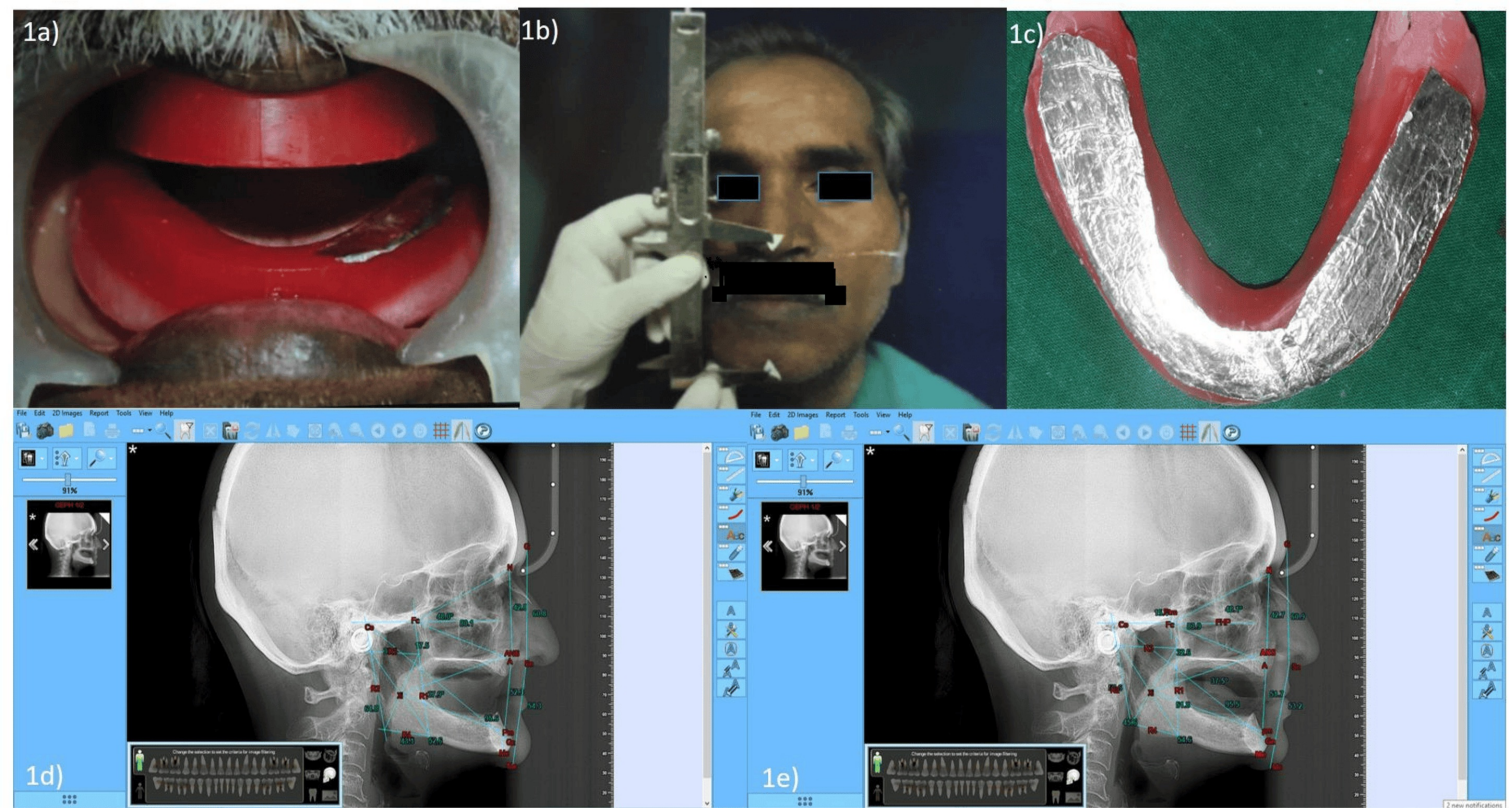
Establishment of the vertical dimension (1a) Occlusal rims in the patient’s mouth. (1b) Vertical jaw relation. (1c) Aluminium foil on the mandibular occlusal rim to easily distinguish the occlusal position. (1d)  Lateral cephalograph at occlusion. (1e) Lateral cephalograph at rest position.

Primary impressions of both arches were made using impression compound (Y-Dents, MDM Corporation, India). Custom trays were fabricated, and border molding was performed with greenstick compound (DPI, India). For recording the final impressions, zinc oxide eugenol paste (DPI, India) was employed. Type III dental stone (Gold Stone, Kalabhai Karson Pvt. Ltd., India) was used to pour the master casts. The record bases were made using self-cure acrylic resin, and occlusal rims were shaped using modeling wax (Figure 1a). The occlusal plane was oriented parallel to the interpupillary line anteriorly and Camper’s plane posteriorly, using a Fox plane for verification. The vertical dimension was clinically determined using the closest speaking space and swallowing methods (Figure 1b). A thin 0.1 mm aluminum foil strip was embedded in the occlusal rim to facilitate radiographic identification of the occlusal plane (Figure 1c).

Radiographic acquisition protocol

Digital lateral cephalograms were acquired using a cephalostat unit (Newtom Giano, Italy). Each subject underwent two radiographs: Without Denture (WOD) recorded at physiologic rest using the phonetic “M” sound method, and With Denture (WD), captured in centric occlusion using the swallowing technique. Standardized imaging parameters included 68 kVp, 5 mA, and 20-second exposure time. Patients were positioned in a natural head posture with ear rods and a head stabilizer. The central X-ray beam was directed perpendicular to the midsagittal plane, and the image receptor was aligned at the level of the external auditory meatus.

Cephalometric analysis

Cephalometric analysis was conducted through digital cephalometric tracing and analysis using dedicated cephalometric software. The anatomical landmarks assessed included both hard and soft tissue points. The hard tissue landmarks comprised Nasion (N), Anterior Nasal Spine (ANS), Menton (Me), Condylion (Co), Gnathion (Gn), Facial Centre (Fc), A-point, Xi-point, and Supra-pogonion (Pm). The soft tissue landmarks assessed were Glabella (G) and Subnasale (Sn). Measurements were derived according to established cephalometric analyses. Specifically, Ricketts and McNamara analyses were utilized for evaluating hard tissue parameters, while the Legan and Burstone method was applied for assessing soft tissue proportions. In both with dentures (WD) and without dentures (WOD) conditions, five key parameters were evaluated (Figures 1d, 1e). These included the proportion of the middle third (N-ANS) and the lower third (ANS-Me) of the face, mandibular length (Co-Gn), the angle of the middle third (N-Fc-A), the angle of the lower third (ANS-Xi-Pm), and the soft tissue proportion (G-Sn/Sn-Me). Each measurement was recorded five times per radiograph to ensure reproducibility and accuracy. The use of a digital platform facilitated the minimization of manual errors and allowed for standardized, repeatable measurements. The values obtained under WOD and WD conditions were subsequently compared to evaluate the correlation between cephalometric and conventional methods in determining the vertical dimension.

Statistical analysis

Data were compiled using Microsoft Excel and analyzed with SPSS software, version 25.0 (IBM Corp., Armonk, NY, USA). Descriptive statistics such as mean, standard deviation, minimum, and maximum values were calculated. To assess the relationship between measurements obtained in WOD and WD conditions, Pearson’s correlation coefficient (r) was applied. A paired t-test was used to evaluate differences between WOD and WD means for each parameter. A p-value of < 0.05 was considered statistically significant. All tests were two-tailed. The analysis was designed to assess the reliability of cephalometric landmarks compared to conventional clinical techniques for evaluating vertical dimension in edentulous patients.

## Results

The findings from Table 1 reveal that both conventional and cephalometric methods showed comparable values in assessing vertical dimension (VD) at rest (WOD) and at occlusion (WD). The conventional method yielded a mean VD of 64.90 mm at rest and 62.10 mm at occlusion, demonstrating a consistent interocclusal clearance (IOC) of approximately 2.80 mm. Similarly, hard tissue measurements (ANS-Me) on lateral cephalograms showed a mean of 65.91 mm at rest and 62.22 mm at occlusion, while soft tissue measurements (Sn-Me) recorded 70.08 mm at rest and 66.98 mm at occlusion. Despite minor variations, all p-values were greater than 0.05, indicating no statistically significant differences between conventional and cephalometric measurements. This supports the conclusion that cephalometric analysis can serve as a reliable adjunctive method in determining vertical dimension.

**Table 1:** Comparison of conventional and cephalometric measurements of vertical dimension (without dentures vs. with dentures) WOD: Without Dentures; WD: With Dentures; SD: Standard Deviation; NS: Not Significant. All measurements are in millimeters (mm) except where specified.

Parameter	Method	Position	N	Min	Max	Mean ± SD (mm)	Mean Difference (mm)	p-value
Vertical Dimension	Conventional	Without Dentures (WOD)	40	52.0	75.0	64.90 ± 5.04	2.80	–
		With Dentures (WD)	40	49.0	72.0	62.10 ± 4.94	–	–
Hard Tissue (Anterior Nasal Spine–Menton)	Cephalometric	Without Dentures (WOD)	40	50.2	85.2	65.91 ± 7.51	2.80	0.100 NS
		With Dentures (WD)	40	48.6	85.7	62.22 ± 7.59	–	0.062 NS
Soft Tissue (Subnasale–Menton)	Cephalometric	Without Dentures (WOD)	40	63.2	80.5	70.08 ± 4.19	3.10	0.281 NS
		With Dentures (WD)	40	55.9	76.9	66.98 ± 4.72	–	0.826 NS

Table 2 further supports this reliability by presenting strong positive correlations between cephalometric measurements taken at rest and occlusion. Notably, the soft tissue proportion (G-Sn/Sn-Me) showed a Pearson correlation coefficient of 0.928, followed by the angle of the middle third of the face (N-Fc-A) at 0.923, and the lower third facial angle (ANS-Xi-Pm) at 0.850. The mandibular length (Co-Gn) also showed a strong correlation (r = 0.808). All correlations were statistically significant (p < 0.001), indicating excellent reproducibility of cephalometric landmarks. These results confirm the potential of cephalometric analysis as a dependable tool for assessing vertical dimension in edentulous patients, particularly when conventional clinical methods may be limited or inconclusive.

**Table 2:** Cephalometric correlation of facial parameters between without dentures (WOD) and with dentures (WD) WOD: Without Dentures; WD: With Dentures; SD: Standard Deviation; N: Nasion; Me: Menton; G: Glabella; Sn: Subnasale; Fc: Facial Centre; Co: Condylion; Gn: Gnathion; Pm: Suprapogonion; NS: Not Significant.

Parameter	WOD Mean ± SD	WD Mean ± SD	Mean Difference	Pearson r	p-value
Skeletal Proportion (Nasion–Menton)	10.45 ± 7.29 mm	9.80 ± 6.75 mm	0.74 mm	–	–
Soft Tissue Proportion (Glabella–Subnasale / Subnasale–Menton)	5.28 ± 3.33 mm	4.54 ± 3.35 mm	0.74 mm	0.928	<0.001
Midface Angle (Nasion–Facial Centre–A-point)	55.09° ± 4.47	53.99° ± 4.55	1.09°	0.923	<0.001
Lower Face Angle (Anterior Nasal Spine–Xi–Suprapogonion)	45.75° ± 3.52	45.65° ± 3.06	0.10°	0.850	<0.001
Mandibular Length (Condylion–Gnathion)	116.05 ± 5.65 mm	113.87 ± 5.42 mm	2.18 mm	0.808	<0.001

## Discussion

The stomatognathic system maintains functional harmony through coordinated interactions among muscles, bones, joints, and teeth [1]. Tooth loss disrupts this balance, necessitating accurate determination of occlusal vertical dimension (OVD) during prosthetic rehabilitation [1,9]. Vertical dimension refers to lower facial height, assessed as vertical dimension at rest (VDR) and at occlusion (VDO) [3]. VDR is a relatively constant, muscle-guided position unaffected by dentition, serving as a reliable reference for establishing VDO [9]. The difference between VDR and VDO defines the freeway space, typically 2-4 mm, which must be individualized. Inadequate freeway space can cause muscle strain and tissue resorption, while excessive space may lead to overclosure and temporomandibular joint dysfunction [3].

OVD loss affects esthetics, phonetics, and masticatory efficiency [10]. A common cause of prosthetic failure is inaccurate VDO determination. Pre-extraction methods such as cephalometry, phonetics, and profile tracing are valuable when prior records exist [11,12]. In their absence, clinicians rely on post-extraction techniques, including the rest position, swallowing, cephalometric analysis, phonetics, facial evaluation, and assessment of existing dentures [3]. Tools like the Willis gauge or digital calipers are used to estimate VDO by subtracting freeway space from VDR, aiding in restoring physiologic facial height.

The mandibular rest position is a clinically reliable reference for determining OVD in edentulous patients due to its relative stability and independence from dentition [13]. It may be assessed using phonetics (e.g., the “M” sound), swallowing, or functional training exercises. Facial esthetics also contribute to OVD evaluation; an ideal dimension presents with relaxed musculature and lightly contacting lips, while deviations may suggest excessive or reduced OVD [4]. However, this method is less dependable in individuals with poor skin tone or lip incompetence. Swallowing-based assessments assume consistent mandibular movement but can vary with technique and individual differences [14].

Other methods include cephalometric analysis, craniofacial landmark measurements (e.g., N-Gn, Sn-Me), and evaluation of existing dentures when suitable [15,16]. Although various approaches, such as rest position, phonetics, facial analysis, and pre-extraction records, are described, none are universally accepted due to patient variability [17]. The method chosen depends on clinical judgment, reproducibility, and individual anatomical factors. Accurate OVD determination is essential for esthetics, comfort, and functional success in complete denture therapy [18].

Cephalometric analysis enables correlation of skeletal and dental relationships with clinical observations and is increasingly applied in prosthodontics for determining OVD [1]. Digital advancements have enhanced cephalometric accuracy and efficiency compared to manual methods. Studies show that computer-assisted cephalometry, when combined with accurate landmark identification, reduces measurement errors [17,19]. In this study, cephalometric landmarks were analyzed using NNT Viewer software in 40 edentulous patients. Lateral cephalograms were taken with and without dentures to evaluate vertical dimension using five parameters: hard and soft tissue proportions, angles of the middle and lower thirds of the face, and mandibular length. These cephalometric parameters were traced and analyzed to assess their reliability and reproducibility.

Niswonger’s method was used for clinical OVD recording and verified with Silverman’s closest speaking space, as recommended by McCord and Grant [20] and others [12,17]. Cephalometric analysis followed the principles of Ricketts, McNamara, and Legan-Burstone. Measurements taken with and without dentures showed a consistent interocclusal clearance (mean difference = 0.745 mm), with a strong Pearson correlation (r = 0.870), indicating reproducibility. The proportion between middle and lower facial thirds (0.8 ± 0.2) remained stable, supporting cephalometry as a reliable adjunct to conventional clinical methods for OVD recording.

In this study, soft tissue proportions were evaluated using Legan and Burstone’s analysis (G-Sn/Sn-Me), which typically follows a 1:1 ratio [21]. A consistent interocclusal clearance (IOC) was observed between the rest (WOD) and occlusion (WD), with a mean difference of 0.74 mm and a strong Pearson correlation (r = 0.928). Similarly, the angle of the middle third (N-Fc-A) and lower third (ANS-Xi-Pm) of the face showed minor differences (1.09° and 0.097°, respectively) and strong correlations (r = 0.923 and r = 0.850), indicating reproducibility. Mandibular length (Co-Gn) showed a mean difference of 2.18 mm and a correlation of r = 0.800. Hard tissue (ANS-Me) and soft tissue (Sn-Me) cephalometric measurements closely matched conventional chin-nose measurements, with no statistically significant differences (p > 0.05), validating the reliability of both approaches.

These findings are supported by prior studies. Qamar et al. reported consistent cephalometric proportions (N-ANS/ANS-Me and G-Sn/Sn-Me) in edentulous patients [22]. Brzoza et al. found similar results in hard (0.8 ± 0.2) and soft tissue (1 ± 0.2) ratios [19]. Orthlieb et al. reported an ANS-Xi-Pm angle mean of 43°, aligning with our result (~45°) [23]. Bhat and Gopinathan confirmed the comparability of conventional and cephalometric VD measurements [17]. Ciftci  et al. [18] observed improved mandibular posture and VD following prosthetic rehabilitation, consistent with the present study. The two-stage cephalometric approach (WOD and WD) using occlusal rims enabled clearer differentiation between physiologic rest and occlusion, enhancing reliability in determining VD.

The present study demonstrated that cephalometric landmarks-specifically, hard tissue (ANS-Me), soft tissue (Sn-Me), the angle of the middle third (N-Fc-A), the angle of the lower third (ANS-Xi-Pm), and mandibular length (Co-Gn)-remained stable between rest (WOD) and occlusal (WD) positions in edentulous patients. These parameters showed strong correlations, with soft tissue landmarks (r = 0.928) being the most reliable, followed by the angle of the middle third, hard tissues, angle of the lower third, and mandibular length. Given their reproducibility, these measurements can effectively aid in determining OVD. Furthermore, integrating the occlusal plane as an additional parameter may enable fabrication of occlusal rims directly from cephalometric data, eliminating the need for traditional jaw relation procedures-a development aligned with the evolution of digital prosthodontics.

This approach shows promise in situations where conventional techniques may be unreliable, such as patients with facial asymmetry, neuromuscular disorders (e.g., Parkinson’s disease, Bell’s palsy), or difficulty maintaining consistent mandibular positioning. The cephalometric method can serve as a dependable adjunct or alternative to conventional OVD determination, especially when soft tissue landmarks are compromised.

However, this study has limitations. Occasional distortion or poor-quality lateral cephalograms may hinder accurate landmark identification and require repeated imaging. Anatomical variations, such as residual ridge resorption, facial asymmetry, or soft tissue sagging in elderly patients, can affect measurement precision. The relatively small sample size (n = 40) also limits the generalizability of the results. Larger multicenter studies are recommended to validate cephalometric landmark reliability across diverse populations. Incorporating cephalometric data into digital tools such as T-scan analysis, 3D facial scans, or CBCT may enhance prosthetic planning. Future research may also explore additional planes, such as the occlusal or esthetic plane, to design occlusal rims without conventional jaw relations. Clinical application in medically compromised or neuromuscularly impaired patients also warrants further investigation.

## Conclusions

The present study demonstrated that cephalometric landmarks, specifically hard tissue and soft tissue proportions, angles of the middle and lower thirds of the face, and mandibular length, remain consistent with and without dentures in edentulous patients. These parameters showed strong correlation and reliability, making them effective adjuncts in determining the OVD. The findings support the use of lateral cephalometry as a reproducible and objective method for establishing OVD, especially in challenging clinical scenarios or when conventional methods are limited. Incorporating cephalometric analysis into digital prosthodontics may offer a simplified and standardized approach for complete denture fabrication.
